# Association of normal weight obesity phenotype with inflammatory markers: A systematic review and meta-analysis

**DOI:** 10.3389/fimmu.2023.1044178

**Published:** 2023-02-27

**Authors:** Nami Mohammadian Khonsari, Fereshteh Baygi, Ozra Tabatabaei-Malazy, Sahar Mohammadpoor Nami, Amir Ehsani, Sasan Asadi, Mostafa Qorbani

**Affiliations:** ^1^ Non-Communicable Diseases Research Center, Alborz University of Medical Sciences, Karaj, Iran; ^2^ Research Unit of General Practice, Department of Public Health, University of Southern Denmark, Odense, Denmark; ^3^ Endocrinology and Metabolism Population Sciences Institute, Non-Communicable Diseases Research Center, Tehran University of Medical Sciences, Tehran, Iran; ^4^ Department of Pediatric, Iran University of Medical Sciences, Tehran, Iran

**Keywords:** NWO, normal weight obesity, inflammation, inflammatory markers, CRP, interleukin

## Abstract

**Background:**

Individuals with normal weight could suffer from obesity based on their body fat percentage (also known as normal weight obesity (NWO)), thus being at risk of significant morbidity and mortality compared to the general population. It seems that inflammatory pathways and chronic inflammation are significant contributors to the pathogenicity of NWO. This study aimed to assess and pool the association of proinflammatory and anti-inflammatory cytokines with NWO.

**Methods:**

In this systematic review and meta-analysis, online international databases (PubMed, Scopus, EMBASE, Web of Science, and Google Scholar) were searched until August 2022. All observational studies with an English full text comparing the mean levels of proinflammatory and anti-inflammatory cytokines (e.g., C-reactive protein (CRP), various types of interleukins (IL) s, tumor necrosis factor-alpha (TNF)) and white blood cell (WBC) count, in subjects with NWO and “normal weight non-obese (NWNO)” were included. Two researchers independently screened, reviewed and assessed the quality of included studies. The remaining articles’ data were extracted post-screening. The heterogeneity between studies was assessed using the I^2^ and Cochran’s Q tests. A random effect model meta-analysis was used to pool the standardized mean difference (SMD) as an effect size.

**Results:**

From the initial 559 studies, 21 and 19 were included in the qualitative and quantitative synthesis, respectively. In the systematic review, 8 studies reported a significant association between various proinflammatory cytokines (CRP, IL_6_, IL_1β_, and TNFα) and NWO. According to random-effect meta-analysis, the association between NWO with CRP (SMD: 0.60, 95% CI: 0.30, 0.91) and IL6 (SMD: 0.90, 95%CI: 0.14, 1.66) was statistically significant. Moreover, the mean level of TNF_α_ in subjects with NWO and NWNO did not differ significantly (SMD: 0.67, 95% CI: -0.36, 1.70).

**Conclusion:**

The findings of this study show that NWO was associated with high levels of CRP and IL6. Therefore, inflammatory pathways may play a role in the pathogenicity of NWO.

## Background

Obesity has been extensively studied as one of the most prominent causes of morbidity and mortality (1, 2). Despite such evaluations in different target populations, new findings still emerge in this topic (3). These findings are particularly important in preventing and treating obesity as its prevalence, morbidity, and mortality are increasing globally (4). It should be noted that lately, morbidity and mortalities attributed to obesity are being seen in individuals who, based on previous definitions of obesity, a body mass index (BMI) above 30 Kg/m2, are not considered obese (5, 6). Hence new definitions and types of obesity have been defined (6).

One of these relatively new definitions regards those with normal BMI values and yet high body fat percentage (6–9). These individuals are regarded as Normal Weight Obese (NWO) (9). Studies indicate that NWOs are at an increased risk of cardiometabolic conditions similar to obese individuals and may suffer from the same morbidity and mortality-related conditions (10–13). Some studies suggest that one contributing factor to this increased risk of cardiometabolic conditions in obesity and NWO could be chronic inflammation, as inflammation has been observed in increased adiposity (14, 15). Despite inflammation being an essential process in the body, chronic inflammation can have adverse cardiometabolic effects, since pro-inflammatory cytokines in a chronic inflammatory status can contribute to the development of atherosclerosis, insulin resistance, type 2 diabetes, hypertension and hypercholesterolemia (14). Since obesity is a chronic condition, the resulting inflammation persists, resulting in chronic inflammation and subsequently the aforementioned conditions (15, 16). Similar to obesity (based on BMI) the association of inflammation with NWO has been highlighted in a review (9). It seems that in NWO the secretions of the adipose tissue itself contributes to inflammation; these studies argue that the underlying cause of this inflammation is the increased fat mass and lipid accumulation resulting in increased oxidative stress, and NF-κB pathway (a major pathway in the innate inflammatory response) activation (9, 17). Although the number of studies addressing the association of NWO and inflammation has been increasing throughout the years (9), no systematic review on the inflammatory aspect of NWO has been published so far. This systematic review and meta-analysis aimed to summarize and pool the association of NWO phenotype with inflammatory markers in published studies.

## Methods

This study was performed according to the Preferred Reporting Items for Systematic Review and Meta-Analysis (PRISMA) checklist (18).

### Search strategy

A comprehensive systematic search was conducted on all available online databases (Scopus, EMBASE, Web of Science, PubMed, and Google Scholar) until August 2022. One of the investigators conducted the search, and another reviewed the findings. Terms such as “normal weight obesity”, “NWO”, “high fat percentage” and their MeSH term equivalents alongside proinflammatory and anti-inflammatory cytokines such as “CRP” “C-reactive protein” and “interleukin” and white blood cell (WBC) count were searched. The entire search (terms and strategy) can be seen as **Supplementary Table 1**. Moreover, reference lists of included studies or reviews were hand-searched to identify more potentially eligible studies.

### Study selection criteria and eligibility

All observational studies with an English full text that assessed the association of proinflammatory and anti-inflammatory cytokines such as C-reactive protein (CRP), various types of interleukins (IL) s, tumor necrosis factor (TNF) alpha with NWO were included in this study. Regardless of their various definitions of NWO (based on fat percentage, waist circumference, etc.), all studies were included. All studies included studies had represented the targeted population and compared them with normal-weight, non-obese (NWNO) individuals. Studies that failed to meet the inclusion criteria were excluded. Furthermore, duplicates, non-peer-reviewed publications, and studies without sufficient information to determine eligibility were excluded.

Two investigators independently carried out the screening process of included studies, including titles, abstracts and full texts. Upon removing the irrelevant entries, the full texts of the remaining articles were assessed. Moreover, to find the missed relevant studies (if any), the reference lists of the included studies were hand-searched as well. Discrepancies were referred to a third investigator for resolution.

### Data extraction strategy

Two investigators separately extracted the data using a pre-designed data extraction sheet. The extracted data were composed of the name of the first author and publication year, the number of participants, age and sex, the definition of normal weight obesity, the studied cytokines, and the outcome as standardized mean difference (SMD) alongside their 95% confidence interval (CI) of the outcomes were extracted as the effect size of dichotomous and continuous respectively. Moreover, discrepancies were referred to a third investigator for resolution.

### Quality assessment

We used the Newcastle-Ottawa Scale for quality assessment. This scale consists of seven items, scoring based on selection, comparability, exposure (case-control studies), and outcome (cohort studies). The total score ranges from 0 to 9 for cohort studies or 0 to 10 for case-control studies and is calculated by summing the scores of each item of this assessment tool (19, 20). We categorized the scores as 0 to 4, 5-6, 7 and above, indicating the studies’ quality (low, middle, and high-quality studies, respectively). Two investigators independently assessed the quality of the studies, and discrepancies were referred to the third investigator.

### Statistical analysis

The I^2^ and Cochran’s Q tests were used to assess the heterogeneity between the studies. A random-effect model was adapted for analyses if the heterogeneity was statistically significant (P-value<0.1). Otherwise, a fixed model was used. The SMDs of the included studies were calculated and pooled as an effect size for NWO association with the mean levels of proinflammatory and anti-inflammatory cytokines. Meta-Analysis was performed for outcomes with at least 3 reports within the studies. If applicable, sub-group analysis was performed for proinflammatory and anti-inflammatory cytokines (stratified by sex, quality and adjustment for confounding variables and type of CRP (high-sensitivity (hs-CRP) and CRP excluding hs-CRP) as well. Egger’s test was adapted to assess publication bias for each inflammatory factor, and trim fill analysis was performed if publication bias was present. STATA (Stata Corporation, College Station, Texas, USA) version 17 was used to analyze the data.

## Results

### Search results

**Figure 1:**
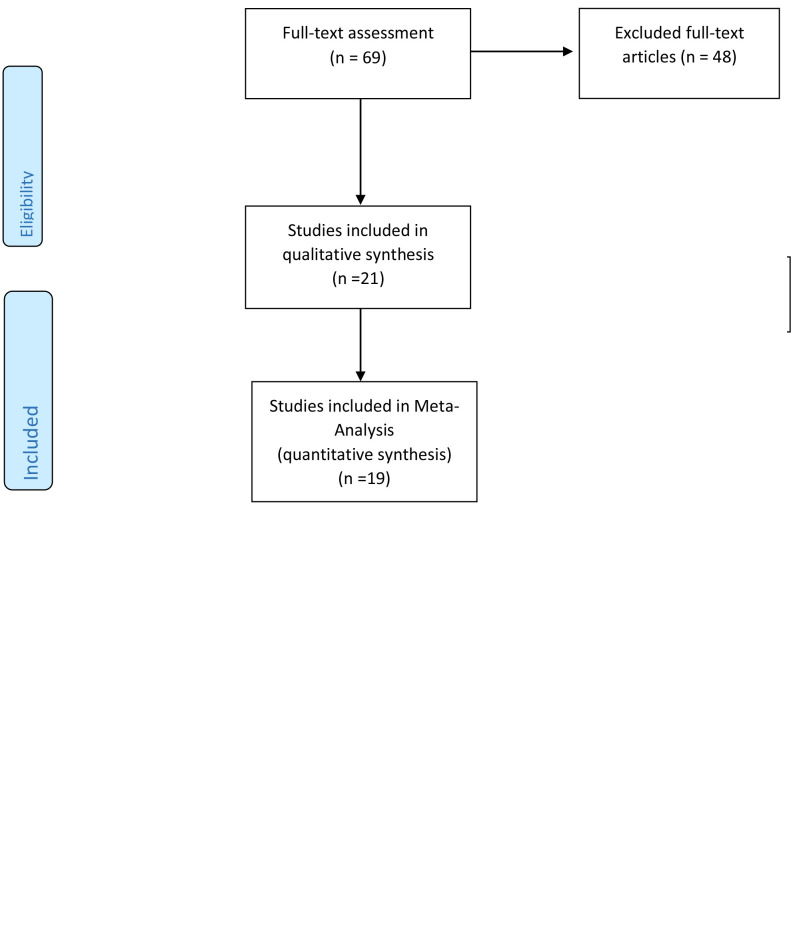
studies search, review and analysis flowchart.

**Figure 1** shows the flowchart of the selection of studies for inclusion in the meta-analysis. From the 559 studies found in the initial search, 263 duplicate studies were removed. Out of 296 remaining articles, 227 irrelevant studies were excluded after titles and abstracts screening. The full texts of the 69 articles were assessed, and 45 studies were excluded due to failing the eligibility criteria. Finally, 21 articles remained in the current systematic review (21–41). However,19 were eligible for inclusion in the meta-analysis (one study reported OR as the effect size (36), and one study exclusively evaluated complement C_3_ (23), which did not reach the minimum number of three studies needed to enter the meta-analysis)

### Study characteristics

The included studies were conducted worldwide (Canada, Iran, Italy, Japan, Poland, South Korea, Spain, Sweden, USA) with a total number of 19,857 participants aged ≥13 years. Twenty studies were conducted on the adult population (age ≥ 18 years), one study was conducted on adolescents aged 13 to 18 (34), and one was conducted exclusively on the elderly aged above 60 (41). Most studies were conducted in Italy (5 studies) and South Korea (4 studies). Canada, Poland, Spain, Sweden and Switzerland had the least number of studies (1 study). The greatest sample size was from a study conducted in the USA (4116 individuals), and the smallest was from Brazil (52 individuals). In most of the included studies, NWO was defined as normal BMI values with a body fat percentage above 30% in women (8 out of 16 reports) and above 25% in men (4 out of 8 reports). These general characteristics are shown in summery in **Table 1**. Three studies (24, 38, 40) had adjusted their findings for possible confounders; the other included studies were not adjusted for any confounding factors.

**Table 1:** General Characteristics of included studies for association of NWO and inflammatory factors.

country	author year	Design	sample size (number)					mean age/age range (year)	NWO definition	Q.A
			Total	M	F	NWNO	NWO			
Brazil	Duque 2021 (21)	Cross-sectional	52	(-)	(-)	29	23	43.3	(-)	3
Canada	J. Shea 2012 (22)	Cross-sectional	653	(-)	(-)	324	329	39.6	BF > 20.8% for men, > 35.0% for women	7
Iran	M. Karkhaneh 2019 (23)	Case-control	70	0	70	30	40	19-39	BF ≥ 30%	4
	Tayefi 2019 (24)	Cross-sectional	2439	(-)	(-)	1311	1128	47	BF > 25% for men, > 30% in women	9
	Amani 2019 (25)	Case-control	90	(-)	(-)	30	30	18-25	BF > 20%	4
Italy	Renzo 2013 (26)	Cross-sectional	47	0	47	17	30	33.6	BF > 30%	3
	Renzo 2010 (27)	Case-control	60	0	60	20	20	20-35	BF > 30%	4
	Renzo 2008 (28)	Case-control	150	0	150	50	50	20-45	BF > 30%	5
	A. Lorenzo 2007 (29)	Case-control	60	0	60	20	20	26	BF > 30%	4
	Renzo 2007 (30)	Case-control	110	0	110	30	40	26	BF > 30%	5
Japan	J. Huang 2018 (31)	Cross-sectional	91	0	91	51	34	20	BF > 30%	5
Poland	W. Kosmala 2012 (32)	Case-control	168	(-)	(-)	95	73	37.8	BF% for every 20 years, from 20 years of age > 19,21,24 for men, 32,33,35 for women	6
south Korea	Y. J. Hyun 2008 (33)	Case-control	50	(-)	(-)	25	25	25-64	visceral fat ≥100 cm2	5
	W. K. Cho 2015 (34)	Cross-sectional	1700	888	812	1266	144	13-18	highest quartile (Q4) of age and sex specific waist to hip ratio	7
	Sohee kim 2015 (35)	Cross-sectional	2078	(-)	(-)	1795	283	53.4	BF ≥ 25.4% for men, ≥ 31.4% for women)	8
	S. Kang 2014 (36)	Case-control	164	(–)	(–)	82	82	54	BF ≥ 23.5% for men, ≥ 29.2% for women	7
Spain	Gómez 2011 (37)	Cross-sectional	3051	838	2213	656	1579	18-80	BF ≥ 25% for men, ≥ 35% for women	8
Sweden	Berg 2015 (38)	Cross-sectional	1471	581	890	1080	266	25-74	BF ≥ 25% for men, ≥ 38% for women	8
Switzerland	P. Marques-Vidal 2010 (39)	Cross-sectional	2301	0	2301	1667	173	54	BF > 38%	8
USA	A. Romero 2010 (40)	Cross-sectional	4116	2031	2085	2054	2062	41.3	BF% ≥23.1% for men, ≥33.3% for women	8
	J. A. Batsis 2013 (41)	Cross-sectional	936	173	763	636	303	> 60	BF > 25% for men, > 35% for women	8

M, male; F, female; NWNO, normal weight non-obese; NWO, normal weight obesity; Q.A, quality assessment (based on the New castle Ottawa scale). (-): not reported/not available.

### Qualitative synthesis

The included studies evaluated the associations of NWO with the mean levels of proinflammatory and anti-inflammatory cytokines (CRP, IFNγ, TNFα, IL_1α_, IL_1β_, IL_2_, IL_6_, IL_8_, IL_10_, IL_12p70_, IL_15_, IL_18_) as well as complement C_3_, and white blood cell count (WBC), compared with normal-weight non-obese (NWNO) individuals; and their effect sizes are shown in **Table 2**. As can be seen, in 11 out of 22 reports of CRP, 2 out of 3 reports of IL_6_, 1 out of 3 reports of TNFα, 2 out of 2 reports of WBC, IL_1α_ and IL_1β_, and 1 out of 1 reports of IL_15_ and complement C_3_, significant differences between NWO and NWNO individuals were observed; with the greatest effect size regarding IL_1β_, SMD: 3.79, 95% CI (2.75-4.83). However, in the two reports evaluating IL_10_ and singular reports evaluating IL_2_, IL_8_, IL_12p70_, IL_18_ and IFNγ, no significant differences between NWO and NWNO individuals were seen. Moreover, one study (36) reported that NWOs, in comparison with NWNOs, have significantly increased odds of vascular inflammation (OR:3.07 95%CI (1.29-7.29)).

**Table 2:** Association of NWO with the mean of inflammatory markers in included studies.

Author, Year	Outcome	Sex	N NWNO	Mean ± SD (N)	N NWO	Mean ± SD (NWO)	SMD NWO/NWNO	95% CI
A. Lorenzo, 2007 (29)	CRP	Female	20	0.4 ± 0.1	20	0.8 ± 0.3	1.75	1.89, 3.62
	IL 1α	Female	20	14.8 ± 1.8	20	26.9 ± 4.5*	3.46	2.69, 4.74
	IL 1β	Female	20	5 ± 2.6	20	15 ± 3.1*	3.43	2.92, 5.07
	IL 2	Female	20	12.3 ± 1.5	20	14.7 ± 3.6	0.85	0.73, 2.12
	IL 8	Female	20	0.9 ± 0.2	20	2.3 ± 0.6*	3.07	1.32, 2.87
	IL 10	Female	20	3.4 ± 0.8	20	3.8 ± 1.3	0.36	0.23, 1.52
	IL 12p70	Female	20	14.2 ± 2.2	20	19.1 ± 3.7	1.58	4.14, 6.85
	IFN γ	Female	20	17.7 ± 4.8	20	25.3 ± 5.3	1.47	2.9, 5.04
A. Romero, 2010 (40)	CRP	Male	1014	3.3 ± 6.36	1017	3.7 ± 6.37*	0.06	(–)
	CRP	Female	1040	3.2 ± 0.32	1045	3.8 ± 0.32*	1.87	(–)
Amani,2019 (25)	CRP	Both	30	2.8 ± 1.8	30	1.6 ± 1.2	-0.77	-0.61, 0.4
Berg, 2015 (38)	CRP	Male	377	0.8 ± 0.99	139	1.4 ± 1.5*	0.52	0.58, 1.11
	CRP	Female	703	0.8 ± 1.35	137	1.2 ± 1.43*	0.29	0.94, 1.49
Duque,2021 (21)	CRP	Both	29	0.1 ± 0.3	23	0.2 ± 0.5	0.25	(–)
Gomez, 2011 (37)	CRP	Male	96	0.9 ± 0.5	371	4.3 ± 9.2*	0.41	0.44, 0.9
	CRP	Female	560	2.1 ± 2.6	1208	4.9 ± 1.95*	1.29	0.21, 0.46
J. A. Batsis, 2013 (41)	CRP	Male	73	0.4 ± 0.25	103	0.6 ± 1*	0.25	(–)
	CRP	Female	563	0.39 ± 0.95	200	0.43 ± 0.56	0.05	(–)
J. Huang 2018 (31)	CRP	Female	51	0.92 ± 0.3	34	1.19 ± 0.58*	0.62	-0.41, 1.29
	TNF α	Female	51	0.65 ± 0.78	34	0.57 ± 0.46	-0.12	-1.25, 0.45
J. Shea, 2012 (22)	CRP	Female	324	2.14 ± 4.22	329	2.42 ± 3	0.08	(–)
M. Karkhaneh, 2019 (23)	C _3_	Female	30	92.79 ± 8.13	40	104.3 ± 15.04*	0.91	(–)
P. Marques-Vidal 2010 (39)	CRP	Female	1667	1.8 ± 0.09	173	1.83 ± 0.27	0.25	2.76, 2.97
Renzo, 2007 (30)	CRP	Female	30	0.5 ± 0.1	40	0.9 ± 0.4	1.28	2.51, 3.94
Renzo, 2008 (28)	CRP	Female	50	0.59 ± 0.1	50	0.9 ± 0.1*	3.08	5.3, 7.21
	IL 6	Female	50	5.45 ± 1.4	50	8.1 ± 3.8*	0.92	0.36, 1.17
Renzo, 2010 (27)	TNF α	Female	20	21.5 ± 5	20	43 ± 10*	2.67	2.92, 5.06
	IL 1α	Female	20	15.2 ± 2	20	27.5 ± 5*	3.17	3.66, 6.14
	IL 1β	Female	20	7.3 ± 2.3	20	17 ± 2.7*	3.79	2.97, 5.14
	IL 6	Female	20	8.2 ± 2.2	20	13.4 ± 1.8*	2.54	2.34, 4.25
	IL 10	Female	20	5.8 ± 0.8	20	6.2 ± 1	0.43	0.59, 1.95
	IL 15	Female	20	6.3 ± 1.1	20	8.7 ± 1.1*	2.14	1.3, 2.84
Renzo, 2013 (26)	CRP	Female	17	0.1 ± 0.17	30	0.4 ± 0.85	0.43	(–)
	TNF α	Female	17	27.51 ± 19.77	30	23.55 ± 11.47	-0.26	(–)
S. Kang, 2014 (36)	CRP	Both	82	1.2 ± 3.2	82	2 ± 2.9	(–)	(–)
Sohee.Kim, 2015 (35)	CRP	Both	1795	1.2 ± 2	283	1.6 ± 2.3*	0.20	(–)
Tayefi, 2019 (24)	CRP	Both	1311	1.28 ± 12.93	1128	1.81 ± 23.3	0.03	(–)
W. K. Cho, 2015 (34)	WBC	Male	662	6 ± 1.34	61	6.4 ± 1.39*	0.30	0.4, 0.74
	WBC	Female	604	5.9 ± 1.25	83	6.1 ± 2.09*	0.14	0.4, 0.79
W. Kosmala, 2012 (32)	CRP	Both	95	1 ± 0.5	73	4 ± 4.2*	1.07	(–)
	IL 6	Both	95	17.2 ± 4.1	73	18.4 ± 6.8	0.22	(–)
	IL 18	Both	95	258 ± 96	73	292 ± 132	0.30	(–)
Y. J. Hyun 2008 (33)	CRP	Both	25	0.23 ± 0.2	25	0.52 ± 0.9	0.44	(–)
	TNF α	Both	25	1.66 ± 0.9	25	2.44 ± 1.65*	0.58	(–)
	IL 6	Both	25	0.96 ± 4.45	25	1.71 ± 0.33*	0.24	(–)

N, number; NWNO, normal weight non-obese; SD, standard deviation; NWO, normal weight obesity; SMD, standardized mean difference; CI, confidence interval; CRP, C-reactive protein; IL, interleukin; IFN, interferon; TNF, Tumor necrosis factor; C, complement; WBC, white blood cell.

The reported outcome values are as follows: CRP: mg/L, ILs, TNF and IFN γ: pg/mL, C _3:_ g/L, WBC: 10 ^9^/L.

*: statistically significant (P-value < 0.05). (-): not reported/not available.

### Quantitative synthesis

**Figure 2:**
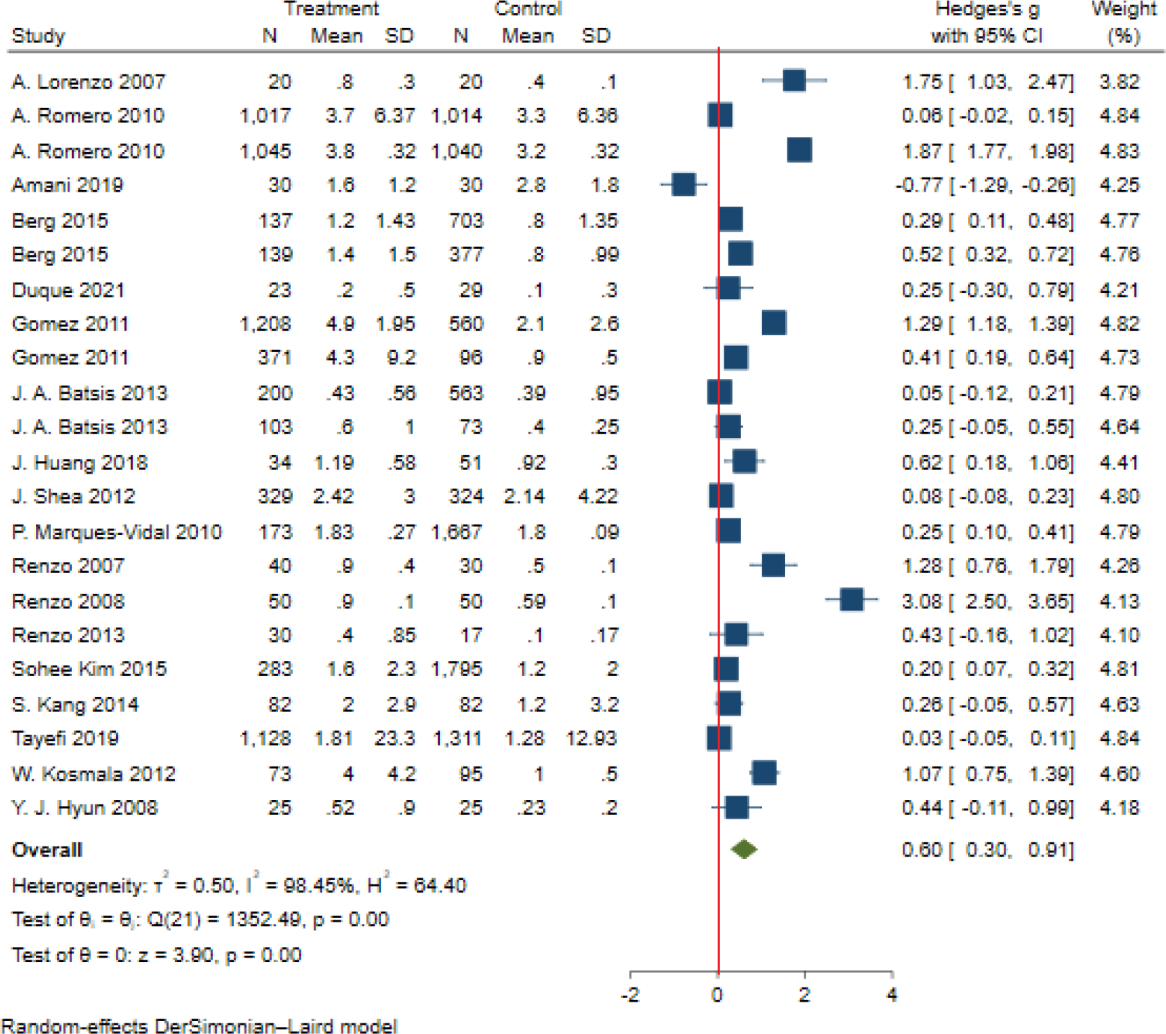
The extracted and overall association between NWO and CRP. (The red line represents null effect).

**Figure 3:**
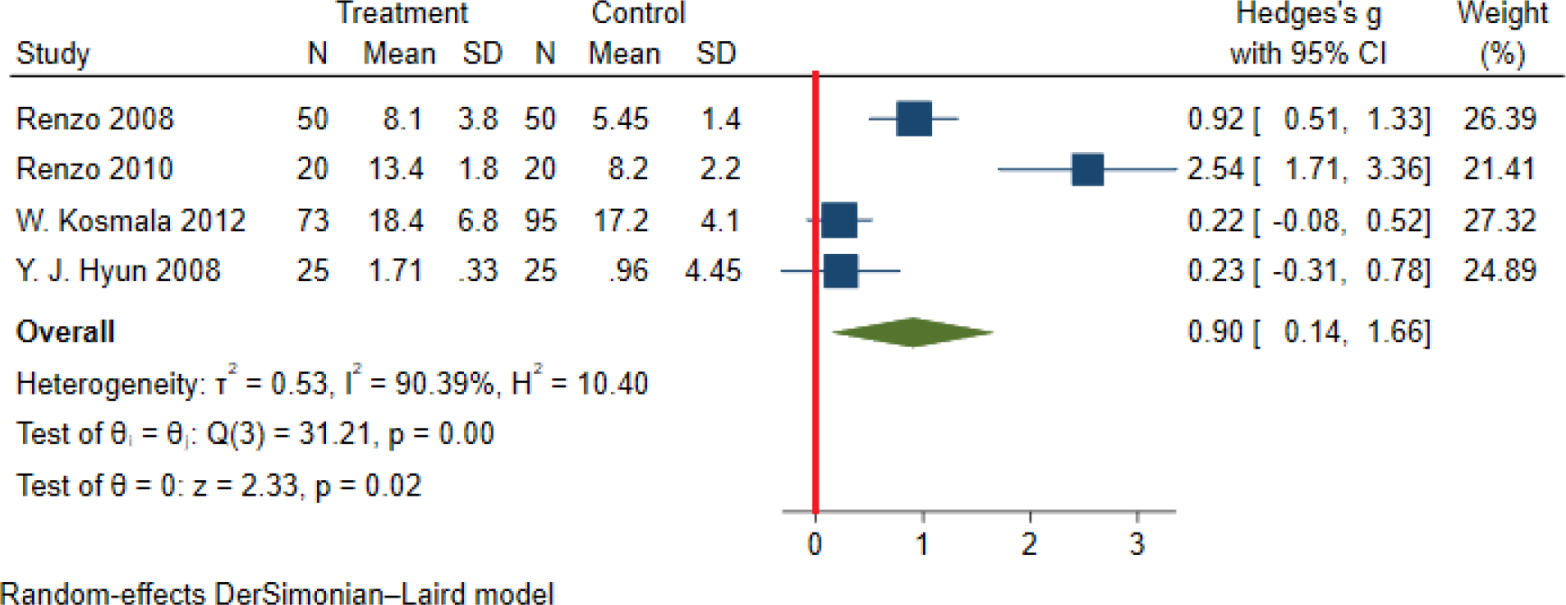
The extracted and overall association between NWO and IL 6. (The red line represents null effect).

**Figure 4:**
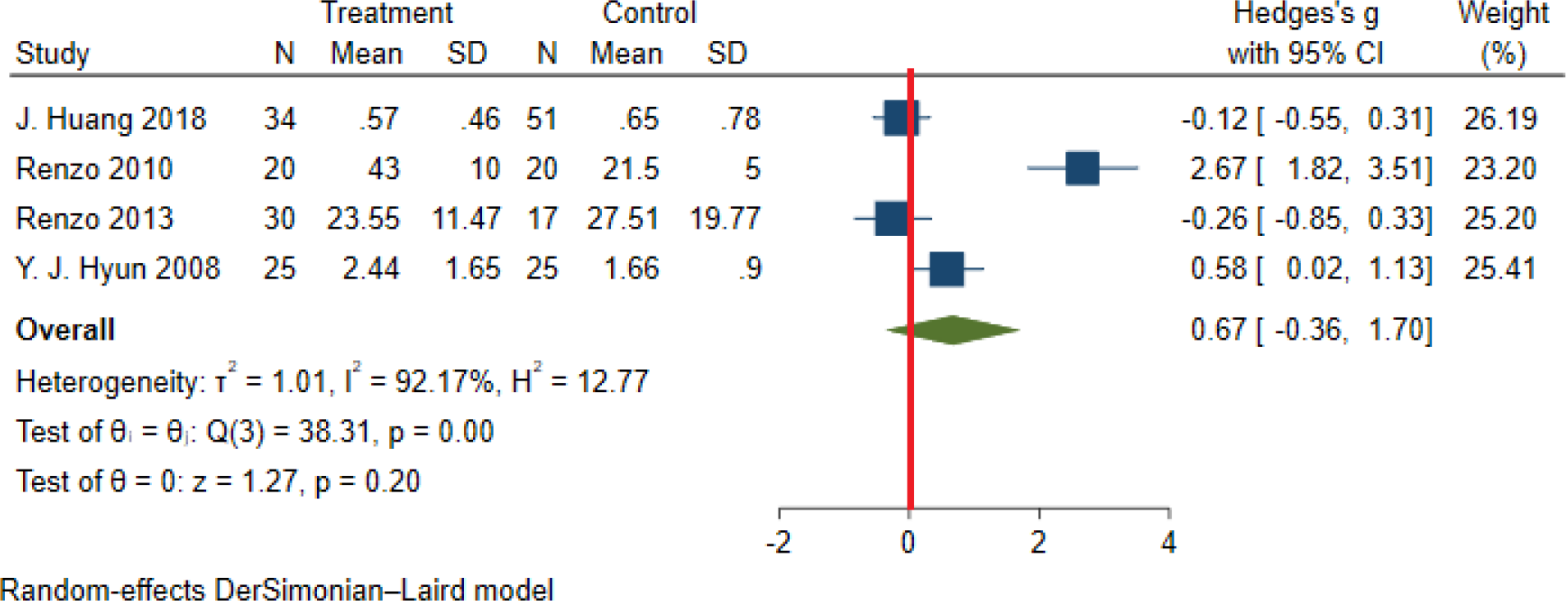
The extracted and overall association between NWO and TNF-α. (The red line represents null effect).

Significant heterogeneity among the studies assessing the association between NWO and CRP, IL_6_ and TNFα was seen (for all associations I^2^: ≥ 88%, P-value < 0.001). The overall association between NWO, and CRP, IL_6_ and TNFα are shown in **Table 3**. Based on the random effect models meta-analysis, the pooled association of CRP (SMD: 0.60, 95% CI: 0.30, 0.91) and IL_6_ (SMD: 0.90, 95%CI: 0.14, 1.66) was significantly higher among NWO individuals compared with NWNOs. TNF-α (SMD: 0.67, 95% CI: -0.36, 1.70) was also higher among NWO individuals; however, this association was not statistically significant. **Figures 2**–**4**, illustrate the included studies and their overall relationships between NWO and CRP, IL_6_ and TNFα, respectively.

**Table 3:** The overall association between NWO, and the means of inflammatory markers.

Variable	N Study	Sample Size	SMD	Heterogeneity		
				I Squared%	Model	**P-Value
Both types CRP^1^						
Overall	18	16492	0.60 (0.30,0.91)*	98.45	Random	< 0.001
By sex						
Male	4	3190	0.31 (0.05,0.56)*	88.87	Random	< 0.001
Female	11	8291	0.97 (0.46,1.48)*	88.7	Random	< 0.001
Both Sexes	7	5011	0.23 (-0.03,0.49)	98.45	Random	< 0.001
By adjustment						
Unadjusted	14	6503	0.65 (0.31,0.98)*	96.24	Random	< 0.001
Adjusted	4	9989	0.50 (-0.14,1.13)	99.47	Random	< 0.001
By quality of the study						
Low quality	4	199	0.39 (-0.56,1.34)	90.65	Random	< 0.001
Medium quality	5	473	1.28 (0.51,2.05)*	92.76	Random	< 0.001
High quality	9	15820	0.43 (0.05,0.80)*	99.01	Random	< 0.001
hs-CRP						
Overall	12	8975	0.65 (0.32,0.99)*	97.34	Random	< 0.001
By sex						
Female	9	5443	0.96 (0.49,1.44)*	97.37	Random	< 0.001
Both Sexes	3	2549	-0.10 (-0.63,0.44)	82.24	Random	< 0.001
By adjustment						
Unadjusted	10	5180	0.78 (0.33,1.22)*	96.91	Random	< 0.001
By quality of the study						
Low quality	3	147	0.45 (-0.95,1.85)	93.77	Random	< 0.001
Medium quality	4	305	1.35 (0.24,2.45)*	94.52	Random	< 0.001
High quality	5	8523	0.41 (-0.01,0.82)	98.34	Random	< 0.001
CRP^2^						
Overall	6	7517	0.60 (-0.12,1.12)	99.17	Random	< 0.001
Both Sexes	4	2462	0.44 (0.03,0.85)*	99.17	Random	< 0.001
Unadjusted	4	1323	0.37 (0.01,0.74)*	0.86.96	Random	< 0.001
High quality	4	7297	0.45 (-0.28,1.18)	99.40	Random	< 0.001
IL6						
Overall	4	358	0.90 (0.14,1.66)*	90.39	Random	< 0.001
TNF α						
Overall	4	222	0.67 (-0.36,1.70)	92.17	Random	< 0.001

N, Number; NWO, Normal Weight Obesity; SMD, Standardized Mean Difference; CI, Confidence Interval; CRP, C-Reactive Protein; IL, Interleukin; TNF, Tumor Necrosis Factor; hs-CRP, high-sensitivity C-reactive protein.

^1^referes to both types of hs-CRP and regular CRP.

^2^referes to CRP excluding hs-CRP.

*P-Values Under 0.05 Were Considered As Statistically Significant.

**For Heterogeneity P-Values Under 0.1 A Fixed Model Was Used.

### Stratified meta-analysis

Based on the random effect model, the means of CRP sub-grouped by sex in males and females were (SMD: 0.31, 95% CI 0.05, 0.56) and (SMD: 0.97, 95% CI 0.31, 0.98), respectively.

The pooled association between NWO and CRP sub-grouped by quality assessment was (SMD: 0.39, 95% CI -0.57, 1.35), (SMD: 1.28, 95% CI 0.51, 2.06), and (SMD: 0.43, 95% CI 0.05, 0.81) for low quality (articles which had a high risk of bias), satisfactory (articles with moderate risk of bias) and high-quality studies (articles with a low risk of bias) respectively. The pooled association between NWO and CRP sub-grouped by study adjustment (whether studies adjusted their findings for confounding variables) was (SMD:0.50 95%CI -0.14,1.13) and (SMD:0.65 95%CI 0.31,0.98) for adjusted and unadjusted studies respectively. The pooled stratified association between NWO and CRP sub-types according to sex, quality assessment and study adjustment are reported in **Table 3**. The association of NWO with hs-CRP was statistically significant (SMD:0.65 95%CI 0.32,0.99), and this association was not statistically significant for CRP excluding hs-CRP (SMD:0.60 95% CI -0.12,1.12).

### Meta-regression

Meta-regression was performed with sex, quality of the studies and study adjustment (whether studies adjusted their findings for confounding variables) as covariates across CRP, IL_6_ and TNFα; no significant results were seen across them except for IL_6_; in which sex and quality of the studies were recognized as possible causes of heterogeneity. (Coefficient: -0.69, P-value: 0.005 and coefficient: -1.61, P-value: 0.001 respectively.)

### Quality assessment

Based on the New-Castle Ottawa scale, six of the included studies were of low quality studies (indicating a high possibility of bias), Five studies had middle quality (with acceptable risk of bias) and ten studies had high quality (with low risk of bias). The overall quality score of the studies can be seen in **Table 1**.

### Publication bias

No publication bias was seen in articles studying the association between NWO and CRP (coefficient: 2.22, P-value: 0.16); however, publication bias was seen in articles assessing NWO and TNF α and IL6 (coefficient: 13.42, P-value: 0.004 and coefficient: 7.62, P-value: 0.017 respectively)

### Trim fill analysis

Sensitivity analysis was performed on studies assessing NWO and TNFα (SMD: 0.67, 95%CI (-0.36, 1.70) and IL6 (SMD: 0.90, 95%CI (0.14, 1.66), indicating that publication bias did not have a substantial effect on the results.

## Discussion

To the best of our knowledge, the current study is the only systematic review and meta-analysis that compared the mean levels of proinflammatory and anti-inflammatory cytokines among NWO and NWNO individuals across the population. Nineteen studies were included in our meta-analysis to address the research questions. And the findings of this study showed that the mean levels of CRP and IL_6_ were significantly higher in NWO individuals compared to the NWNO individuals.

Although inflammation is one of the body’s essential processes, chronic inflammation is not desirable (42). As chronic inflammation could cause damage to the inflamed site, resulting in metabolic dysregulation, homeostatic mechanisms alteration and even result in some diseases (anemia, various tissue damages, malnutrition, and autoimmune diseases) (42–44). It should be kept in mind that chronic inflammation itself can cause progressive atherosclerosis and cardiovascular diseases through various mechanisms (43, 45). In this regard, although IL_6_ is an essential modulator of the immune system and has a wide range of biological activities such as modulating immune responses (46), inflammation and hematopoiesis (46, 47); its increase has been associated with renal injury (48), autoimmune conditions (e.g. rheumatoid arthritis and Crohn’s disease), increased risk of cardiovascular disease and increased mortality due to cardiovascular diseases (49, 50). Similarly, it seems that CRP is an important regulator of inflammation and not just a marker (51). Elevated levels of CRP have been seen in autoimmune conditions (e.g. rheumatoid arthritis) and infections and inflammation (51). Furthermore, elevated CRP has been associated with cardiovascular conditions (52), atherothrombosis (53) and atherosclerosis (54). As studies suggest, elevated levels of both IL_6_ and CRP have been associated with cardiovascular diseases (55). Hence it seems that alongside the increased adiposity which is a significant risk factor for cardiovascular diseases (56), the increased IL_6_ and CRP associated with NWO could have a significant effect on atherosclerosis and cardiovascular diseases as well. Thus by reducing inflammation with various methods (proper nutrition, exercise, etc.), the cardiovascular risk imposed by NWO may be reduced to some degree (8, 16). Moreover, to reduce the comorbidities of NWO, the public should be educated on various types of obesity and the risks that they impose; they should know that the BMI system has its flaws, and a normal BMI does not indicate the absence of obesity.

It should be kept in mind that CRP could be affected by many environmental factors; thus, IL levels could be more suitable for association assessment and research purposes (57).

### Limitations and strength

Despite this study being the aggregated data of all studies on inflammation and NWO with a more precise estimation, the majority of the included studies were not adjusted for potential confounders and had a relatively small sample size; hence studies with a greater population and proper adjustments (e.g. age, sex, underlying diseases, inflammatory and infectious conditions prior to testing, etc) are needed to properly evaluate the association of IL_6_, CRP and TNFα with NWO; and since many of the included studies were unadjusted the findings of these studies, should be interpreted with caution. Moreover, only IL_6_, CRP and TNFα had enough studies making meta-analysis rational. More studies on other aspects of inflammation are needed to evaluate their association with NWO as well.

## Conclusion

The present study highlighted the significant association of NWO with CRP and IL6 and showed that these cytokines were significantly higher in NWO individuals compared to the NWNO individuals, pertaining to the presence of some degrees of inflammation among NWOs. Regarding the aligned effect of inflammation and adiposity in the progression of cardiovascular diseases and, most importantly, the flaws in the current BMI system, using other measures alongside BMI, and implementing preventive measures to reduce adiposity and inflammation is needed. Moreover, more studies on inflammatory markers in NWO individuals are needed to understand their association better.

## Data availability statement

The original contributions presented in the study are included in the article/**Supplementary Material**. Further inquiries can be directed to the corresponding authors.

## Author contributions

NM and MQ, FB, designed the study. NM and SM searched the databases. NM and SA screened and extracted the data. NM and FB analyzed the data and prepared the results. NM and MQ wrote the paper. FB, OT-M and AE edited and revised the manuscript. All other authors contributed to the article and approved the submitted version.

## References

[1] Finer N . Medical consequences of obesity. Medicine (2015) 43(2):88–93. doi: 10.1016/j.mpmed.2014.11.003

[2] Ortega Francisco B Lavie Carl J Blair Steven N . Obesity and cardiovascular disease. Circ Res (2016) 118(11):1752–70. doi: 10.1161/CIRCRESAHA.115.306883 27230640

[3] He C Zhang M Li J Wang Y Chen L Qi B . Novel insights into the consequences of obesity: A phenotype-wide mendelian randomization study. Eur J Hum Genet (2022) 30:540–6. doi: 10.1038/s41431-021-00978-8 PMC9091238 34974530

[4] Lim HJ Xue H Wang Y . Global trends in obesity. In: Meiselman H. Eds. Handbook of Eating and Drinking (Cham: Springer (2020). doi: 10.1007/978-3-030-14504-0_157

[5] García-Hermoso A Agostinis-Sobrinho C Camargo-Villalba GE González-Jiménez NM Izquierdo M Correa-Bautista JE . Normal-weight obesity is associated with poorer cardiometabolic profile and lower physical fitness levels in children and adolescents. Nutrients (2020) 12(4):1171. doi: 10.3390/nu12041171 32331411 PMC7230357

[6] Khonsari NM Khashayar P Shahrestanaki E Kelishadi R Nami SM Heidari-Beni M . Normal weight obesity and cardiometabolic risk factors: A systematic review and meta-analysis. Front Endocrinol (2022) 13:857930. doi: 10.3389/fendo.2022.857930 PMC8987277 35399938

[7] Anoop S Kapoor N . Normal-weight obesity: A hidden pandemic. In: Faintuch J. Faintuch S. Eds. Obesity and Diabetes Cham: Springer (2020). doi: 10.1007/978-3-030-53370-0_26

[8] Franco LP Morais CC Cominetti C . Normal-weight obesity syndrome: diagnosis, prevalence, and clinical implications. Nutr Rev (2016) 74(9):558–70. doi: 10.1093/nutrit/nuw019 27473199

[9] Oliveros E Somers VK Sochor O Goel K Lopez-Jimenez F . The concept of normal weight obesity. Prog Cardiovasc Dis (2014) 56(4):426–33. doi: 10.1016/j.pcad.2013.10.003 24438734

[10] Xu S Ming J Jia A Yu X Cai J Jing C . Normal weight obesity and the risk of diabetes in Chinese people: A 9-year population-based cohort study. Sci Rep (2021) 11(1):1–8. doi: 10.1038/s41598-021-85573-z 33731778 PMC7969601

[11] Shirasawa T Ochiai H Yoshimoto T Nagahama S Kobayashi M Ohtsu I . Associations between normal weight central obesity and cardiovascular disease risk factors in Japanese middle-aged adults: A cross-sectional study. J Health Population Nutr (2019) 38(1):1–7. doi: 10.1186/s41043-019-0201-5 PMC6918653 31849344

[12] Perini W Kunst AE Snijder MB Peters RJG van Valkengoed IGM . Nutrition , metabolism & cardiovascular diseases ethnic differences in metabolic cardiovascular risk among normal weight individuals : Implications for cardiovascular risk screening. HELIUS study. Nutr Metab Cardiovasc Dis (2019) 29(1):15–22. doi: 10.1016/j.numecd.2018.09.004 30467070

[13] Kim S Joo HJ Shim W-J Lee J . Normal weight obesity and metabolic syndrome risk in Korean adults: 5-year longitudinal health checkup study. Circulation (2018) 138(Suppl_1):A13448–A.

[14] Unamuno X Gómez-Ambrosi J Rodríguez A Becerril S Frühbeck G Catalán V . Adipokine dysregulation and adipose tissue inflammation in human obesity. Eur J Clin Investig (2018) 48(9):e12997. doi: 10.1111/eci.12997 29995306

[15] Karczewski J Śledzińska E Baturo A Jończyk I Maleszko A Samborski P . Obesity and inflammation. Eur Cytokine Netw (2018) 29(3):83–94. doi: 10.1684/ecn.2018.0415 30547890

[16] Ferrante A . Obesity-induced inflammation: A metabolic dialogue in the language of inflammation. J Internal Med (2007) 262(4):408–14. doi: 10.1111/j.1365-2796.2007.01852.x 17875176

[17] Elks CM Francis J . Central adiposity, systemic inflammation, and the metabolic syndrome. Curr Hypertens Rep (2010) 12(2):99–104. doi: 10.1007/s11906-010-0096-4 20424938

[18] Page MJ McKenzie JE Bossuyt PM Boutron I Hoffmann TC Mulrow CD . The PRISMA 2020 statement: An updated guideline for reporting systematic reviews. J Clin Epidemiol (2021) 134:178–89. doi: 10.1016/j.jclinepi.2021.03.001 33789819

[19] Lo CK-L Mertz D Loeb M . Newcastle-Ottawa Scale: comparing reviewers’ to authors’ assessments. BMC Med Res methodol (2014) 14(1):1–5. doi: 10.1186/1471-2288-14-45 24690082 PMC4021422

[20] Wells GA Shea B O’Connell D Peterson J Welch V Losos M . The Newcastle-Ottawa scale (NOS) for assessing the quality of nonrandomised studies in meta-analyses. Oxford (2000).

[21] Duque AP Barbosa IM Lins AS de Jesus FG Araújo CF Id N . Abstract P028: Increased cardiometabolic risk and autonomic function alterations in normal weight obesity. Circulation (2021) 143(Suppl_1):AP028–AP. doi: 10.1161/circ.143.suppl_1.P028

[22] Shea JL King MT Yi Y Gulliver W Sun G . Body fat percentage is associated with cardiometabolic dysregulation in BMI-defined normal weight subjects. Nutr Metab Cardiovasc Dis (2012) 22(9):741–7. doi: 10.1016/j.numecd.2010.11.009 21215604

[23] Karkhaneh M Qorbani M Ataie-Jafari A Mohajeri-Tehrani MR Asayesh H Hosseini S . Association of thyroid hormones with resting energy expenditure and complement C3 in normal weight high body fat women. Thyroid Res (2019) 12:9. doi: 10.1186/s13044-019-0070-4 31666810 PMC6813955

[24] Tayefi M Tayefi B Darroudi S Mohammadi-Bajgiran M Mouhebati M Heidari-Bakavoli A . There is an association between body fat percentage and metabolic abnormality in normal weight subjects: Iranian large population. Trans Metab Syndrome Res (2019) 2(1):11–6. doi: 10.1016/j.tmsr.2019.08.001

[25] Amani R Parohan M Jomehzadeh N Haghighizadeh MH . Dietary and biochemical characteristics associated with normal-weight obesity. Int J Vitamin Nutr Res (2019) 89(5-6):331–6. doi: 10.1024/0300-9831/a000477 30856081

[26] Di Renzo L Sarlo F Petramala L Iacopino L Monteleone G Colica C . Association between -308 G/A TNF-alpha polymorphism and appendicular skeletal muscle mass index as a marker of sarcopenia in normal weight obese syndrome. Dis Mark. (2013) 35(6):615–23. doi: 10.1155/2013/983424 PMC3830785 24285913

[27] Di Renzo L Galvano F Orlandi C Bianchi A Di Giacomo C La Fauci L . Oxidative stress in normal-weight obese syndrome. Obesity (2010) 18(11):2125–30. doi: 10.1038/oby.2010.50 20339360

[28] Di Renzo L Bertoli A Bigioni M Gobbo VD Premrov MG Calabrese V . Body composition and -174G/C interleukin-6 promoter gene polymorphism: association with progression of insulin resistance in normal weight obese syndrome. Curr Pharm Des (2008) 14(26):2699–706. doi: 10.2174/138161208786264061 18991689

[29] De Lorenzo A Del Gobbo V Premrov MG Bigioni M Galvano F Di Renzo L . Normal-weight obese syndrome: early inflammation? Am J Clin Nutr (2007) 85(1):40–5. doi: 10.1093/ajcn/85.1.40 17209175

[30] Di Renzo L Bigioni M Del Gobbo V Premrov MG Barbini U Di Lorenzo N . Interleukin-1 (IL-1) receptor antagonist gene polymorphism in normal weight obese syndrome: relationship to body composition and IL-1 alpha and beta plasma levels. Pharmacol Res (2007) 55(2):131–8. doi: 10.1016/j.phrs.2006.11.002 17174563

[31] Huang J Fukuo K Yoshino G Kazumi T Basetty C Huang Y . (2018). Body composition and biochemical characteristics of normal weight obesity in Japanese young women with different physical activities, in: 2018 IEEE international conference on bioinformatics and biomedicine (BIBM), Madrid, Spain, pp. 1480–3. doi: 10.1109/BIBM.2018.8621153

[32] Kosmala W Jedrzejuk D Derzhko R Przewlocka-Kosmala M Mysiak A Bednarek-Tupikowska G . Left ventricular function impairment in patients with normal-weight obesity: Contribution of abdominal fat deposition, profibrotic state, reduced insulin sensitivity, and proinflammatory activation. Circulation: Cardiovasc Imaging. (2012) 5(3):349–56. doi: 10.1161/CIRCIMAGING.111.969956 22407472

[33] Hyun YJ Koh SJ Chae JS Kim JY Kim OY Lim HH . Atherogenecity of LDL and unfavorable adipokine profile in metabolically obese, normal-weight woman. Obesity (2008) 16(4):784–9. doi: 10.1038/oby.2007.127 18239579

[34] Cho WK Kim H Lee HY Han KD Jeon YJ Jung IA . Insulin resistance of normal weight central obese adolescents in Korea stratified by waist to height ratio: results from the Korea national health and nutrition examination surveys 2008–2010. Int J Endocrinol (2015) 2015:158758. doi: 10.1155/2015/158758 26257779 PMC4519535

[35] Kim S Kyung C Park JS Lee S-P Kim HK Ahn CW . Normal-weight obesity is associated with increased risk of subclinical atherosclerosis. Cardiovasc diabetol (2015) 14(1):1–9. doi: 10.1186/s12933-015-0220-5 25990248 PMC4488951

[36] Kang S Kyung C Park JS Kim S Lee S-P Kim MK . Subclinical vascular inflammation in subjects with normal weight obesity and its association with body fat: an 18 f-FDG-PET/CT study. Cardiovasc Diabetol (2014) 13(1):1–12. doi: 10.1186/1475-2840-13-70 24708764 PMC3994236

[37] Gómez-Ambrosi J Silva C Galofré JC Escalada J Santos S Millán D . Body mass index classification misses subjects with increased cardiometabolic risk factors related to elevated adiposity. Int J Obes (2011) 36(2):286–94. doi: 10.1038/ijo.2011.100 21587201

[38] Berg C Strandhagen E Mehlig K Subramoney S Lissner L Björck L . Normal weight adiposity in a Swedish population: how well is cardiovascular risk associated with excess body fat captured by BMI? Obes Sci Pract (2015) 1(1):50–58. doi: 10.1002/osp4.4 PMC5049492 27721982

[39] Marques-Vidal P Pécoud A Hayoz D Paccaud F Mooser V Waeber G . Normal weight obesity: Relationship with lipids, glycaemic status, liver enzymes and inflammation. Nutrition Metab Cardiovasc Dis (2010) 20(9):669–75. doi: 10.1016/j.numecd.2009.06.001 19748248

[40] Romero-Corral A Somers VK Sierra-Johnson J Korenfeld Y Boarin S Korinek J . Normal weight obesity: A risk factor for cardiometabolic dysregulation and cardiovascular mortality. Eur Heart J (2010) 31(6):737–46. doi: 10.1093/eurheartj/ehp487 PMC2838679 19933515

[41] Batsis JA Sahakyan KR Rodriguez-Escudero JP Bartels SJ Somers VK Lopez-Jimenez F . Normal weight obesity and mortality in united states subjects >/=60 years of age (from the third national health and nutrition examination survey). Am J Cardiol (2013) 112(10):1592–8. doi: 10.1016/j.amjcard.2013.07.014 23993123

[42] Pahwa R Goyal A Jialal I . Chronic inflammation. StatPearls [Internet]. Treasure Island (FL): StatPearls Publishing (2021). Available from: https://www.ncbi.nlm.nih.gov/books/NBK493173/. 29630225

[43] Hotamisligil GS . Inflammation and metabolic disorders. Nature (2006) 444(7121):860–7. doi: 10.1038/nature05485 17167474

[44] Straub R . The origin of chronic inflammatory systemic diseases and their sequelae. Elsevier Inc (2015). doi: 10.1016/C2014-0-04588-1

[45] Lopez-Candales A Burgos PMH Hernandez-Suarez DF Harris D . Linking chronic inflammation with cardiovascular disease: from normal aging to the metabolic syndrome. J Nat Sci (2017) 3(4):e341. 28670620 PMC5488800

[46] Kimura A Kishimoto T . IL-6: Regulator of Treg/Th17 balance. Eur J Immunol (2010) 40:1830–5. doi: 10.1002/eji.201040391 20583029

[47] Mihara M Hashizume M Yoshida H Suzuki M Shiina M . IL-6/IL-6 receptor system and its role in physiological and pathological conditions. Clin Sci (Lond). (2012) 122(4):143–59. doi: 10.1042/CS20110340 22029668

[48] Su H Lei CT Zhang C . Interleukin-6 signaling pathway and its role in kidney disease: An update. Front Immunol (2017) 8:405. doi: 10.3389/fimmu.2017.00405 28484449 PMC5399081

[49] Patterson CC Smith AE Yarnell JW Rumley A Ben-Shlomo Y Lowe GD . The associations of interleukin-6 (IL-6) and downstream inflammatory markers with risk of cardiovascular disease: the Caerphilly study. Atherosclerosis (2010) 209(2):551–7. doi: 10.1016/j.atherosclerosis.2009.09.030 19836021

[50] Volpato S Guralnik JM Ferrucci L Balfour J Chaves P Fried LP . Cardiovascular disease, interleukin-6, and risk of mortality in older women: the women's health and aging study. Circulation (2001) 103(7):947–53. doi: 10.1161/01.cir.103.7.947 11181468

[51] Sproston NR Ashworth JJ . Role of c-reactive protein at sites of inflammation and infection. Front Immunol (2018) 9:754. doi: 10.3389/fimmu.2018.00754 29706967 PMC5908901

[52] Wilson AM Ryan MC Boyle AJ . The novel role of c-reactive protein in cardiovascular disease: risk marker or pathogen. Int J Cardiol (2006) 106(3):291–7. doi: 10.1016/j.ijcard.2005.01.068 16337036

[53] Devaraj S Singh U Jialal I . The evolving role of c-reactive protein in atherothrombosis. Clin Chem (2009) 55(2):229–38. doi: 10.1373/clinchem.2008.108886 PMC2662846 19095731

[54] Libby P Ridker PM . Inflammation and atherosclerosis: role of c-reactive protein in risk assessment. Am J Med (2004) 116 Suppl 6A:9S–16S. doi: 10.1016/j.amjmed.2004.02.006 15050187

[55] Nadrowski P Chudek J Skrzypek M Puzianowska-Kuźnicka M Mossakowska M Więcek A . Associations between cardiovascular disease risk factors and IL-6 and hsCRP levels in the elderly. Exp Gerontol. (2016) 85:112–7. doi: 10.1016/j.exger.2016.10.001 27729238

[56] Poirier P . Adiposity and cardiovascular disease: are we using the right definition of obesity? Eur Heart J (2007) 28(17):2047–8. doi: 10.1093/eurheartj/ehm321 17673449

[57] Abbas AK Lichtman AH Pillai S . Cellular and molecular immunology e-book. Elsevier Health Sciences (2021) eBook ISBN: 9780323757508 Paperback ISBN: 9780323757485.

