# Supplementary material for: Association of normal weight obesity phenotype with inflammatory markers: A systematic review and meta-analysis
Source: Front Immunol. 2023 Feb 27;14:1044178. doi: 10.3389/fimmu.2023.1044178 (PMC10010388; doi:10.3389/fimmu.2023.1044178)
Supplement: Supplementary file 1 [file Table_1.docx]

| **PubMed** |
| --- |
| ("Normal Weight Obesity"[Title/Abstract] OR "Central Obesity"[Title/Abstract] OR "Obesity Phenotype"[Title/Abstract] OR "NWO"[All Fields] OR "Regional Body Fat"[Title/Abstract] OR "normal weight"[Title/Abstract] OR "Normal Weight Phenotype"[Title/Abstract] OR "Normal Body Mass Index"[Title/Abstract]) AND ("inflammation"[Title/Abstract] OR "inflammatory"[All Fields] OR "CRP"[All Fields] OR "IL"[All Fields] OR "Interleukin"[All Fields] OR "ESR"[All Fields] OR "TNF"[All Fields] OR "IFN"[All Fields]) OR "complements"[All Fields]) OR "complement"[All Fields]) OR "WBC"[All Fields]) AND ("NWNO"[Title/Abstract] OR "non obese"[All Fields] OR "without obesity"[All Fields]) |
| **Scopus** |
| ( TITLE-ABS-KEY ( Normal Weight Obesity )  OR  TITLE-ABS-KEY ( *"* Normal BMI Obesity *"* )  OR  TITLE-ABS-KEY ( *"* Central Obesity *"* )  OR  TITLE-ABS-KEY ( *"* Obesity Phenotype *"* )  OR  TITLE-ABS-KEY ( *"* Regional Body Fat *"* )  OR  TITLE-ABS-KEY ( *NWO* )) AND ( TITLE-ABS-KEY (inflammation ) OR TITLE-ABS-KEY ( " inflammatory " ) OR TITLE-ABS-KEY ( " CRP " ) OR TITLE-ABS-KEY ( " IL " ) OR TITLE-ABS-KEY ( " Interleukin " ) OR TITLE-ABS-KEY (ESR ) OR TITLE-ABS-KEY (“TNF” ) OR TITLE-ABS-KEY (“IFN” ) OR TITLE-ABS-KEY (“complements” ) OR TITLE-ABS-KEY (“complement” ) OR TITLE-ABS-KEY (“WBC” )) AND ( TITLE-ABS-KEY (NWNO ) OR TITLE-ABS-KEY ( " non obese " ) OR TITLE-ABS-KEY ( " without obesity " ) |
| **ISI/WOS** |
| (TS= (Normal Weight Obesity) OR TS= ("Normal BMI Obesity ") TS= ("Central Obesity ") OR TS= ("Obesity Phenotype ") OR TS= (Regional Body Fat) OR TS= ("NWO")) AND (TS= (inflammation) OR TS= ("inflammatory ") TS= ("CRP ") OR TS= ("IL ") OR TS= (Interleukin) OR TS= ("ESR ") OR TS= ("TNF ") OR TS= ("IFN ") OR TS= ("WBC ") OR TS= ("complements ") OR TS= ("complement ")) AND (TS= (NWNO) OR TS= ("non obese") TS= ("without obesity ")  Indexes=SCI-EXPANDED, SSCI, CPCI-S, CPCI-SSH, ESCI Timespan=All years |

**Supplementary Table 1: Search strategy**
